# Uriticaria and angioderma after ingestion of grapes

**DOI:** 10.1186/2045-7022-1-S1-P91

**Published:** 2011-08-12

**Authors:** Ana Célia Costa, Pedro Morais Silva, Maria Conceição Santos, Manuel Pereira Barbosa

**Affiliations:** 1 Santa Maria Hospital - CHLN, Immunoallergology Department, Lisbon, Portugal; 2 Molecular Medicine Institute/Lisbon Medical School, Clinical Immunology Unit, Lisbon, Portugal

## Background

Grape (*Vitis vinifera*) allergy is considered rare and usually found in association with pollinosis. Recent publications identified Vit v 1, a grape lipid transfer protein (LTP), as a major allergen that is sometimes involved in severe reactions. Other minor allergens, like a protein homologous to the cherry thaumatin-like protein may also play a role in cross-reactivity reactions.

## Case report

We report the case of a 28-year-old female who developed acute generalized urticaria and facial angioedema one hour after ingesting grapes of several varieties. The reaction was treated at Emergency Room level with parenteral administration of corticoids and anti-histamines. She previously ingested grapes and other fresh fruits with no reaction and denied rhinitis complaints. Skin prick tests with a large battery of aeroallergens, including latex, were positive to peach LTP, peach, apple, and plum but were negative with grape commercial extract. Prick by prick procedure performed with the pulp and peel of a variety of red and white grapes yielded positive results, as well as with fresh cherry. Specific IgE (kUA/l; ImmunoCAP®, Phadia) were present for peach (1.16), peach LTP (1.79), apple (1.17), plum (1.36) and cherry (0.8) and were negative for grape.

## Conclusions

Although infrequent, grape allergy may present with severe reactions. In this case, a LTP seems to be the major allergen responsible for the patient's reaction. Prick by prick procedure should be performed in patients with a grape allergy suspicion because commercial extracts may not be completely reliable.

